# 3T MRI in evaluation of asbestos-related thoracic diseases – preliminary results

**DOI:** 10.2478/v10019-010-0027-7

**Published:** 2010-05-24

**Authors:** Janez Podobnik, Igor Kocijancic, Viljem Kovac, Igor Sersa

**Affiliations:** 1 Institute of Radiology, University Clinical Centre Ljubljana, Ljubljana, Slovenia; 2 Department of Radiation Oncology, Institute of Oncology Ljubljana, Ljubljana, Slovenia; 3 Jožef Stefan Institute, Ljubljana, Slovenia

**Keywords:** 3T, magnetic resonance imaging, asbestos-related thoracic disease, malignant pleural mesothelioma

## Abstract

**Background:**

3T high-field magnetic resonance imaging (MRI) scanners have recently become available for the clinical use and are being increasingly applied in the field of whole-body imaging and chest imaging as well. The aim of this study was to evaluate the diagnostic potential of 3 T MRI as a complementary imaging modality to CT in detecting the pathological changes of asbestos-related thoracic diseases.

**Patients and methods:**

Fifteen patients with the asbestos-related thoracic disease were scheduled for 3T MRI. Five had a benign form of the disease and 10 had malignant pleural mesothelioma (MPM). From the patients with a benign form of the disease their last CT examination in digital form was acquired and patients with MPM were scheduled for CT examination with contrast media. The protocol of MR imaging consists of T2-weighted cardiac-gated breath-hold turbo spin echo (TSE) sequences in coronal, sagittal and axial plane and T1-weighted cardiac-gated breath-hold TSE black blood in axial plane. In T2-weighted sequences in axial plane, fat saturation was also used. CT examinations were obtained with the administration of the contrast medium from lung apices to the lower end of the liver. Images of 5 mm (mediastinum window) and 3 mm (lung window) in axial plan were reconstructed. MRI signal intensity of lesions and adjacent muscles on Syngo MultiModality Work Place were measured.

**Results:**

Compared to muscles pleural plaques appeared hypo-intense to iso-intense on *T1* weighted images (in 100%) and also hypo-intense on *T2* fs-weighted images (in 100%). MPM appeared inhomogeneous hypo-intense to iso-intense on *T1*-weighted and hyperintense on *T2* fs-weighted images in all patients (100%).

**Conclusions:**

These preliminary results pointed out that MRI was equal or even better compared with CT examination for detecting possible malignant potential of pleural changes in the asbestos-related pleural disease, using signal intensity measurements of *T2* fs-weighted images. The 3T MRI enabled the accurate determination of chest pathology and it could be used for imaging of patients with the asbestos-related thoracic disease. MRI is particularly valuable because a patient is not exposed to the harmful radiation which is important if imaging methods are used repeatedly, like in screening programs or in monitoring of treatment results. This finding turned us to propose 3T MRI imaging technique as a non-ionizing imaging method for the follow-up of patients with the isolated pleural form of the asbestos-related disease.

## Introduction

Asbestos is a generic term applied to a variety of naturally formed hydrated silicates, which were used because of their heat resistance properties. Asbestos-related thoracic diseases are benign pleural effusions, pleural plaques, diffuse pleural thickening, rounded atelectasis, asbestosis, mesothelioma, and lung cancer. Asbestos is a mainly cause for malignant mesothelioma, much more important than the potential other causes as Simian virus.^1^ The incidence of malignant mesothelioma is expected to peak between 2010 and 2030 in industrialized countries despite the regulatory restriction during the 1980s and 1990s.^2^

CT is a gold standard tool for the detection of the asbestos-related thoracic disease although recent studies revealed that MRI was superior in the detection of the invasive growth of malignant pleural mesothelioma (MPM) in diaphragm and endothoracic fascia or the detection of single chest wall focus.^3^ A high radiation dose in repeated CT examination and the use of iodine contrast media in patients with renal disease, diabetes and known allergy must be considered.

3T high-field MRI scanners have recently become available for the clinical use and are now increasingly being applied in the field of the whole body imaging and the thoracic imaging as well. Due to the fact that the signal-to-noise ratio is directly related to the static magnetic field strength the spatial resolution can be increased and the examination time can be shortened.^4^

MRI of the lungs is limited because of physical and physiological factors such as low proton density, susceptibility effect, and respiratory movements as well as cardiac and vascular pulsation. Susceptibility artefacts, magnetic field distortion and motion artefacts are increased in high-field MRI. However, the signal loss from normal lung parenchyma due to the susceptibility effect and the theoretically increased signal from solid changes may result in a higher contrast between the normal lungs and pathological changes in high-field MRI.^5^,^6^

To reduce susceptibility artefacts the turbo spin-echo (TSE) with a short echo spacing sequence has been recommended.^7^ To avoid respiratory movements the breath-hold technique in inspiration with the examination time under 20 seconds should be used^6^ and to prevent cardiac and vascular pulsation artefacts ECG triggering must be applied. To avoid additional movements phase array coil should be properly attached with belt.^8^

The aim of our study was to evaluate the diagnostic performance of 3T MRI for detection and characterization of asbestos-related thoracic diseases in comparison to CT.

## Patients and methods

Fifteen patients with the asbestos-related thoracic disease (ARTD) were scheduled for 3T MRI. Five had a benign form of the disease and 10 had MPM. From the patients with a benign form of ARTD their last CT examination in digital form, which was not older than one month, was acquired and patients with MPM were scheduled for CT examination with contrast media.

All patients with the benign form of ARTD were males and occupationally exposed to asbestos. The median age was 66 years in range from 53 to 76 years. In patients with MPM were 6 males and 4 females. The median age of this group was 62 years in range from 50 to 75 years. Only 3 males were occupationally exposed to asbestos, in others the history of the environmental exposure was described.

The study was approved by the national medical ethic committee of the Republic of Slovenia. A written consent was obtained from all patients.

### MRI studies

MR studies were performed with the Trio team system (Siemens, Erlangen, Germany) equipped with the gradient system with a maximum gradient amplitude 40 mT/m and slew rate of 200 mT/m/ms. A matrix body coil with 6 elements in combination with a spine coil was used.

The protocol of MR imaging consists of *T2-*weighted cardiac-gated breath-hold TSE sequences in coronal, sagittal and axial plane and *T1*-weighted cardiac-gated breath-hold TSE black blood in axial plane. In *T2*-weighted sequences in axial plane Spectral pulse for saturation the signal from fat (SPIR - fs) was also used.

*T2*-weighted TSE with following parameters were performed:
  Long TR (repetition time) – depends on heart rate (two cardiac cycles were used)
    - TE (echo time) 100 ms
    - Slice thickness 5 mm with 1 mm gap
    - Turbo factor 29 (Echo Train Length 5)
    - Field of view between 350 to 400 mm (depends on the patient size)
    - Matrix size 208 x 320 with interpolation
    - Parallel imaging factor (iPAT) 2
    - Acquisition time <20 seconds.
*T1*-weighted TSE with following parameters were performed:
  - Short TR – depends on heart rate (one cardiac cycles were used)
  - TE (echo time) 28 ms
  - Slice thickness 5 mm with 2.5 mm gap
  - Turbo factor 9 (Echo Train Length 11)
  - Field of view between 350 to 400 mm (depends on the patient size)
  - Matrix size 106 x 256 with interpolation
  - Parallel imaging factor (iPAT) 2
  - Acquisition time <14 seconds.

The examination time takes from 25 to 30 minutes depending on the patient’s cooperation.

### CT examinations

CT examinations were obtained with Somatom 16 or Definition scanners (Siemens, Erlangen, Germany) with the administration of the contrast medium using a power injector with 2 ml/s flow. CT scans with 120 kV and 100 mAs were performed from lung apices to the end of the liver. Kernel B31f medium smooth for mediastinal window (W: 350, C: 35) and B80f ultra sharp for the lung window (W: 1600, C: −600) were used. Images of 5 mm (mediastinal window) and 3 mm (lung window) in axial plan were reconstructed.

### Measurement of MRI signal intensity

MRI signal intensity of lesions on Syngo MultiModality Work Place were measured to establish their hyper intensity or hypo intensity compared to the signal intensity of muscles.

On *T1* and *T2* fs-weighted images in axial plane the circular region of interests (ROI) with area expanse from 0.3 to 0.4 cm^2^ were drawn. The region with the pronounced artefact was avoided. Data were collected and arranged regarding to the characteristics of the lesion.

MRI and CT examinations were assessed by two radiologists experienced in chest imaging.

## Results

**FIGURE 1 A, B:**
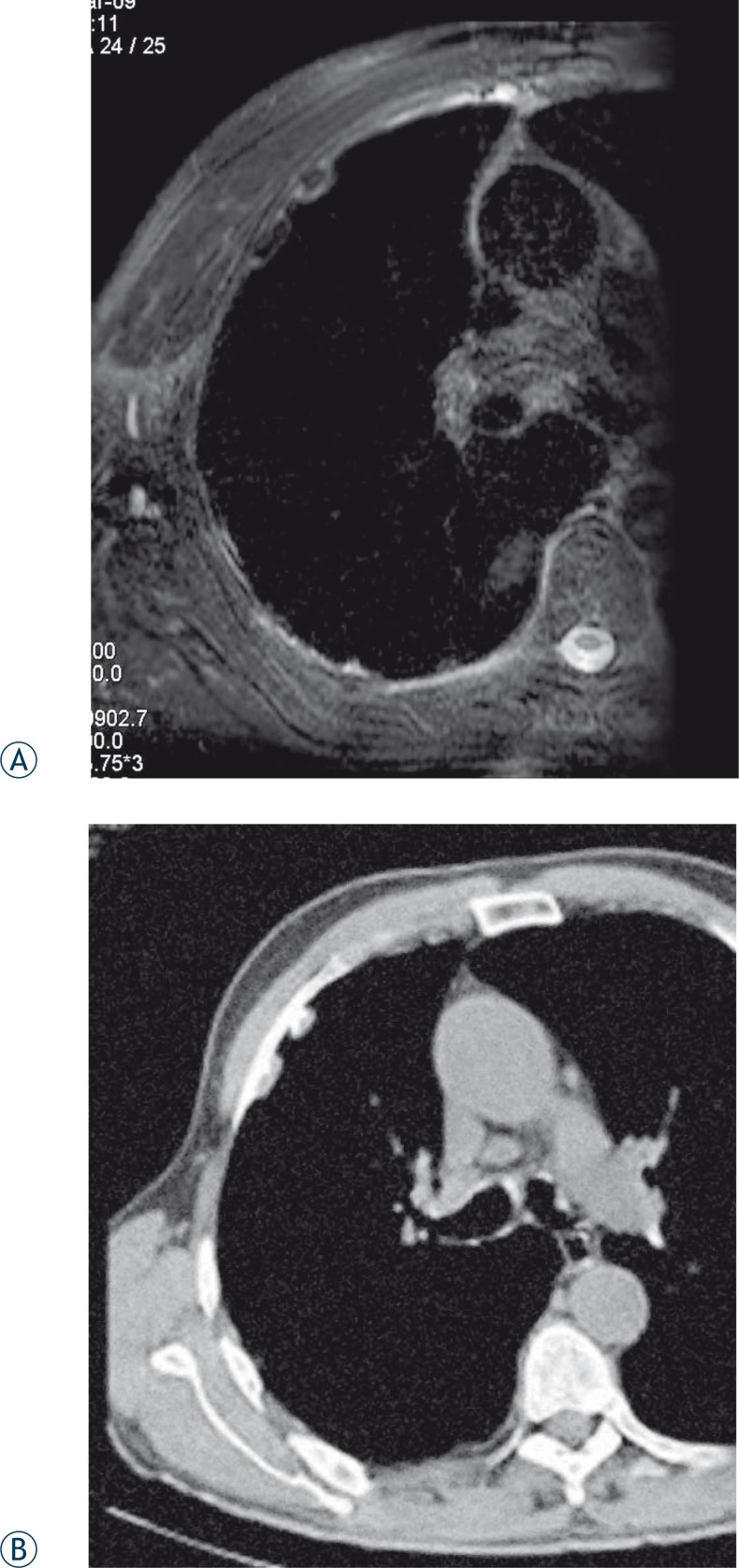
MRI and CT images of the same patient. (A) *T2* fs-weighted MRI image in axial plane shows benign hypo-intense pleural plaques with hyper-intense rim. (B) On CT image calcification on the lateral side of the plaques are more delineated than on MRI images.

Compared to muscles benign pleural plaques appeared hypo-intense on *T2* fs-weighted images and on *T1* weighted images. In some plaques a hyper-intense rim between the hypo-intense plaque centre and lung parenchyma on *T2* fs- weighted images was found (Figure 1). A diffuse pleural thickening was more distinctive on MRI than on CT images.

**FIGURE 2 A, B:**
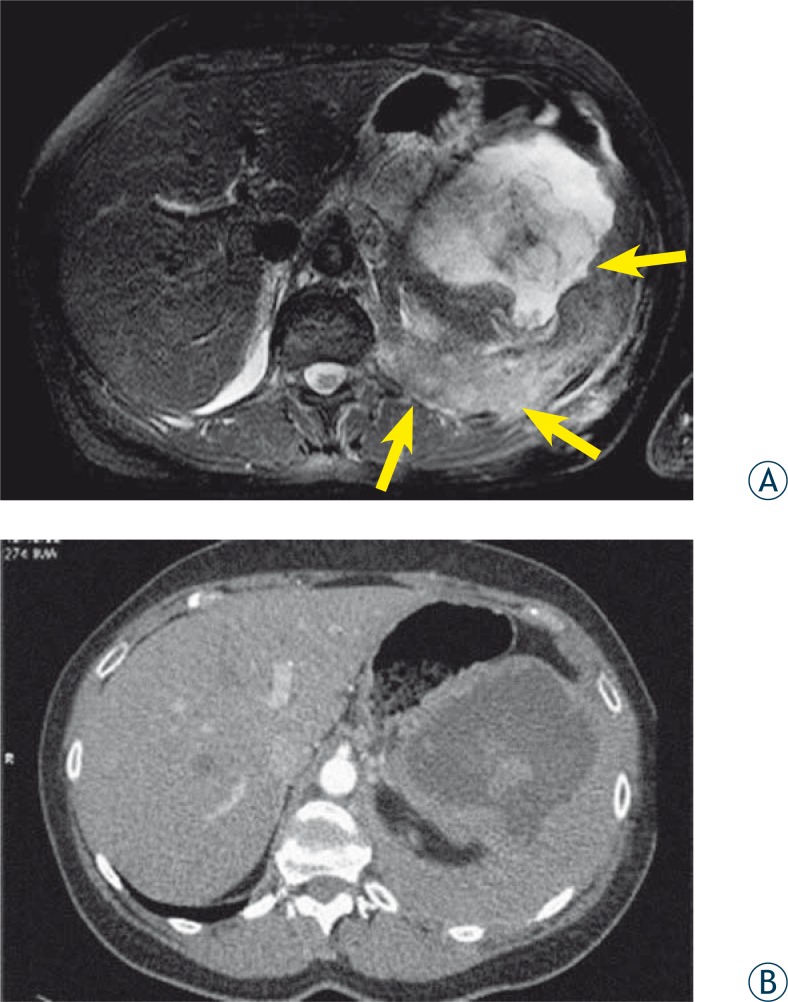
MRI and CT images of the same patient. (A) *T2* fs-weighted MRI image in axial plane shows chest wall and diaphragmatic invasion and the extent of the tumor in abdominal cavity (arrows). (B) Contrast enhanced CT image on the same position shows worse contrast resolution compared to MRI.

MPM appeared inhomogeneous hypo-intense to iso-intense on *T1*-weighted and hyperintense on *T2* fs-weighted images. SPIR was used to saturate the signal from the fat and consequently a high signal compared to the muscle was only found in malignant lesions. In our study 3T MRI shows the extent of the tumour and accompanying pleural solid and fluid components with the greater accuracy compared to CT examination (Figure 2).

**FIGURE 3:**
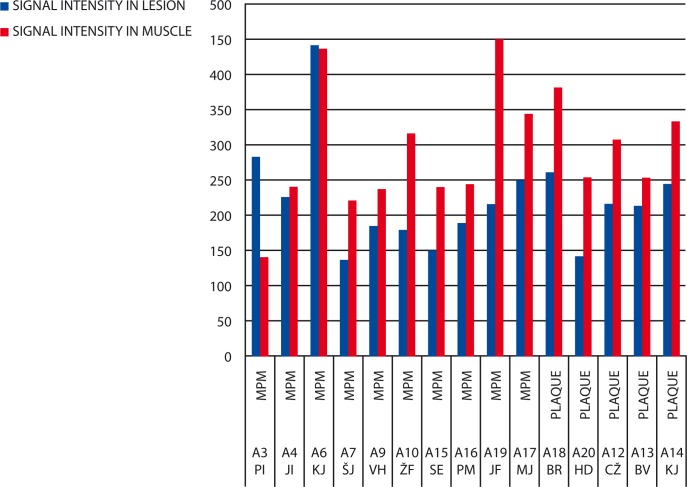
Signal intensity measurement on T1 fs-weighted images. MPM = malignant pleural mesothelioma

Results of signal intensity measurements on *T1*-weighted images (Figure 3) have shown that the MR signal from benign pleural plaques compared to muscles was hypo-intense to iso-intense in all patients (100%). In eight patients with MPM (80%) the measured MR signal was hypo-intense, iso-intense in one patient (10%) and hyper-intense in one patient (10%).

**FIGURE 4:**
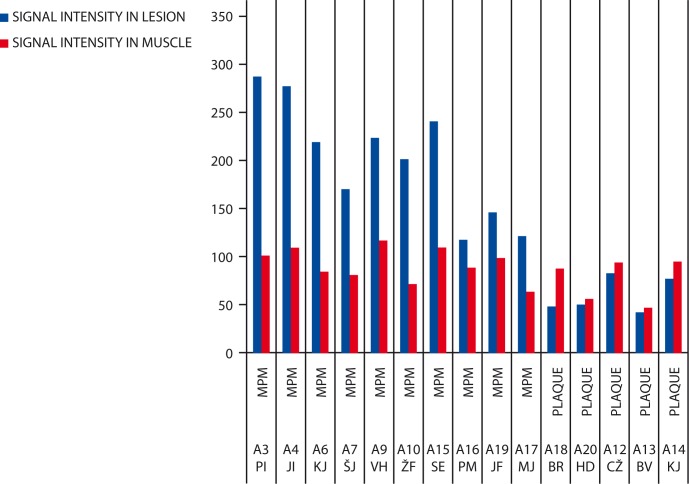
Signal intensity measurement on T2 fs-weighted images. MPM = malignant pleural mesothelioma

Results of measurements on *T2* fs-weighted images (Figure 4) have shown that the MR signal from benign plaques compared to muscles was hypo-intense in all patients (100%) and hyper-intense in patients with MPM (100%).

## Discussion

The benefit of 3T high field MR scanners which have recently become available for the clinical use is higher signal-to-noise ratio. For this reason the examination time can be shortened and the spatial resolution can be improved.^4^ These facts are the reason that MRI is expanding on the field of chest imaging and becoming comparable imaging modality to CT and proposed as a valuable alternative to certain patient groups.^9^

The most significant advantage of MRI of MPM is its excellent contrast resolution of soft tissues. Recent studies have demonstrated that MRI is superior to CT in evaluating of MPM invasive growth in diaphragm and abdominal cavity, invasion of endothoracic fascia and mediastinal structures.^3^,^10^ Falaschi *et al.*^11^ analyzed the potential usefulness of MR signal intensity in differentiating the malignant from the benign pleural disease and concluded that the hypo-intense signal in pulse sequences with long TR is a reliable predictive sign of the benign disease. In our study the intensity of the signal measured in benign pleural plaques compared to muscles on *T1*-weighted images were hypo-intense to iso-intense. On *T2* fs-weighted images the signal intensity from MPM compared to muscles was hyper-intense in all patients.

CT using high resolution protocols is superior to MRI in imaging of parenchymal and early inter-stitial involvement in ARTD, but MRI achieved a comparable interobserver agreement in detecting pleural plaques compared to CT and a higher interobserver agreement in revealing other pleural pathologies.^2^,^7^ In the same article it is also stated that a hyper-intense rim of benign asbestos plaques next to lung parenchyma on *T2* weighted images possibly correlate to reduced plaque collagen fibrils and that diffuse pleural thickenings are more pronounced on MRI than on CT. In our small group of patients with asbestosis (altogether 5 patients) we found that imaging of the asbestos pleural disease with 3T MR protocols was comparable with CT imaging showing almost the same extent and nature (calcified and non-calcified) of pleural plaques, but statistically significant conclusions on this statement needs to be confirmed on the larger number of patients.

The incidence of ARTD is rising but the pattern of ARTD has been changing due to the intensity of exposures. The incidence of MPM will probably increase at least twenty years after the interdiction of asbestos.^13^ This fact requires the evaluation of screening programs for the detection of early stage malignant changes in a high risk group. MRI is a very suitable imaging method in repeatedly screening programs because the patient is not exposed to a harmful radiation. MRI can be also used in monitoring patients treated with chemotherapy.

The results of our preliminary study show that CT is still a gold standard in imaging patients with thoracic diseases, also because the large number of CT scanners is available. 3T MRI is a promising method which can be nowadays used as a complementary method.
